# What Are the Impacts of GLP‐1 Drugs in Patients Needing Head/Neck Surgery?

**DOI:** 10.1002/lary.70341

**Published:** 2025-12-25

**Authors:** Chelsea Gelboin‐Burkhart, Liam Gallagher, Bridget Ebert, Samir S. Khariwala

**Affiliations:** ^1^ Department of Otolaryngology—Head and Neck Surgery University of Minnesota Minneapolis Minnesota USA

## 1 Background

Recent advancements in treatment of metabolic diseases have led to increasing use of Glucagon‐Like Peptide‐1 receptor agonists (GLP‐1RAs) which target multiple organ systems and lead to improved glucose control and management of obesity. In the GI tract, GLP‐1 signals lead to increased gastric emptying time, and side effects can include nausea and vomiting which raises the risk of aspiration when undergoing anesthesia. There have been reports of pulmonary aspiration undergoing procedural sedation or general anesthesia and a number of organizations have provided data and general recommendations regarding the perioperative management of these medications. In this review, we gather the data on the perioperative use of GLP‐1RAs and provide some insights for the practicing Otolaryngologist to guide preoperative planning and patient counseling.

## 2 Literature Review

In 2024, multiple organizations, including representation from anesthesia, gastroenterology, and bariatric surgery, published a clinical practice guidance for perioperative management of GLP‐1RAs [1]. They highlighted the importance of shared decision making between the patient, surgeon, anesthesia team, and prescribing physician to weigh the risks of aspiration associated with delayed gastric emptying against the metabolic benefit. The patients at highest risk include those in the escalation phase of treatment (initial 4–8 weeks or following dose increases), at higher maintenance doses, and who are experiencing gastrointestinal side effects or have comorbidities that slow GI motility (i.e., Parkinson's disease). In low‐risk patients, continuing GLP‐1RA therapy was recommended without interruption. For higher‐risk patients, they recommend implementing a clear liquid diet for 24 h prior to surgery, consideration of rapid‐sequence induction, or routine gastric point‐of‐care ultrasound to assess gastric contents on the day of surgery. If held, daily‐delivered GLP‐1As should be withheld the day of surgery, and weekly GLP‐1RAs should be held 1 week prior to surgery. This consensus statement was based on a number of studies investigating the effect of GLP‐1RAs on gastric emptying and postoperative glucose control and perioperative observational studies.

A systematic review of randomized control trials investigated the effect of GLP‐1 analogues on a variety of parameters including gastric emptying, appetite, and taste [2]. Gastric emptying was assessed via the acetaminophen absorption method in 190 patients. In one study, 56 patients with obesity were treated with Liraglutide (a daily long‐acting GLP‐1RA) ramped up over the course of 6 weeks and a significant delay in gastric emptying was found between groups at week 6. In another group of 49 patients, two doses of Liraglutide were tested (1.8 mg vs. 3.0 mg) with a significant reduction of gastric emptying in the 3.0 mg dosed group (23% reduction). The 1.8 mg dosed group had a 13% reduction in gastric emptying; however, this was not statistically significant. In two studies in which patients received IV infusions of GLP‐1RAs, gastric emptying was slower during the infusion. Semaglutide (a weekly long‐acting GLP‐1RA) did not consistently impede gastric emptying in the studies included. This includes 1 study in which 28 patients were escalated from 0.25 mg to 0.5 mg for 12 weeks and another in which 72 adults were treated for 20 weeks and escalated to 2.4 mg/week. It is notable that these findings may be due to the acetaminophen absorption methodology as other studies have demonstrated semaglutide‐related delayed gastric emptying via scintigraphy and gastric ultrasound.

The safety of preoperative GLP‐1RA use in patients undergoing elective surgery was evaluated in a recent systematic review by Kamarajah et al. evaluating 97,059 patients across 21 studies [3]. They identified no increase in postoperative complications including aspiration pneumonia, delayed gastric emptying, and postoperative vomiting. However, this differs from other studies identifying a dose‐dependent relationship between GLP‐1RA and GI side effects, and a higher incidence of aspiration pneumonia in GLP‐1RA users. This study was primarily comprised of bariatric, orthopedic, or general surgical patients and the timing of preoperative cessation was not noted.

Among patients with poorly controlled diabetes, shorter‐acting GLP‐1RAs have been used postoperatively as a method to control hyperglycemia which may pertain to cases necessitating strict glucose control, that is, necrotizing fasciitis or acute invasive fungal sinusitis. Hult et al. conducted a systematic review investigating the role of GLP‐1RAs and other similar medications in the peri‐operative or intensive care setting [4]. They included 19 single‐center randomized control trials; in 12 studies, lower blood glucose levels were demonstrated, and in 3 studies, reduced insulin was needed for equivalent glucose control. They found no difference in hypoglycemia events between groups, and no increase in post‐operative nausea or vomiting was noted except when liraglutide was delivered the night prior to surgery (13% vs. 0%). The patients in this study were either mechanically ventilated or had undergone cardiac surgery.

Due to preclinical evidence of increased risk of medullary thyroid carcinoma (MTC) from long term exposure to GLP‐1RAs in rodents, a family history of MTC or multiple endocrine neoplasia type 2 (MEN2) is a contraindication to prescribing GLP‐1RAs. To further investigate this risk and other potential effects on thyroid disorders, Hu et al. performed a meta‐analysis of published RCTs [5]. They analyzed 45 RCTs and calculated risk ratios for the occurrence of thyroid cancer (all subtypes), hyper and hypothyroidism, thyroiditis, thyroid mass, and goiter. GLP‐1RA use had no effects on any of the above; however, there was an association with an increased risk of overall thyroid disorders (28% increased relative risk). While this and similar studies have not found an association between GLP‐1RA use and MTC, these studies are limited in statistical power; thus, additional investigations are needed to further elucidate the risk of thyroid cancer in human subjects.

## 3 Best Practice Summary

While controversy remains surrounding perioperative GLP‐1 management, the evidence suggests that the increased risk of delayed gastric emptying and aspiration may be stratified by dose, phase of treatment, and ongoing symptoms. In low‐risk patients, GLP‐1RAs may be safely continued through the perioperative period. In higher‐risk patients, modification of preoperative guidelines, including 24‐h clear liquid diet, rapid‐sequence intubation, and point‐of‐care gastric ultrasound, should be considered. For surgeries in which the outcome is affected by postoperative vomiting (i.e., skull base resections, laryngectomy), and patients undergoing monitored anesthesia care without airway protection, a lower threshold for medication interruption may be warranted; however, this needs further investigation. When indicated, the consensus recommends holding once‐daily dosages for 1 day and once‐weekly dosages for 1 week prior to surgery, and postoperative hyperglycemia should be expected and managed accordingly. If held longer than this preoperatively, diabetic patients should discuss bridging therapy to manage hyperglycemia with their endocrinologist. Short‐acting GLP‐1RAs may be considered to improve glucose control postoperatively while hospitalized in select patients. While we have extrapolated data from other surgical disciplines, additional studies are needed to investigate GLP‐1RA use in otolaryngologic patients, which remains a critical gap in the literature.

## Funding

The authors have nothing to report.

## Conflicts of Interest

The authors declare no conflicts of interest.

## Data Availability

Data sharing not applicable to this article as no datasets were generated or analyzed during the current study.
